# Multiplexed detection of respiratory pathogens with a portable analyzer in a “raw-sample-in and answer-out” manner

**DOI:** 10.1038/s41378-021-00321-7

**Published:** 2021-11-23

**Authors:** Nan Li, Minjie Shen, Jiajia Liu, Li Zhang, Huili Wang, Youchun Xu, Jing Cheng

**Affiliations:** 1 State Key Laboratory of Membrane Biology, Department of Biomedical Engineering, School of Medicine, Tsinghua University, Beijing, 100084 China; 2 National Engineering Research Center for Beijing Biochip Technology, Beijing, 102206 China; 3 Center for Precision Medicine, West China Hospital, Sichuan University, Chengdu, 610041 China

**Keywords:** Electrical and electronic engineering, Chemistry

## Abstract

Coronavirus disease 2019 (COVID-19) has emerged, rapidly spread and caused significant morbidity and mortality worldwide. There is an urgent public health need for rapid, sensitive, specific, and on-site diagnostic tests for severe acute respiratory syndrome coronavirus 2 (SARS-CoV-2) infection. In this study, a fully integrated and portable analyzer was developed to detect SARS-CoV-2 from swab samples based on solid-phase nucleic acid extraction and reverse transcription loop-mediated isothermal amplification (RT-LAMP). The swab can be directly inserted into a cassette for multiplexed detection of respiratory pathogens without pre-preparation. The overall detection process, including swab rinsing, magnetic bead-based nucleic acid extraction, and 8-plex real-time RT-LAMP, can be automatically performed in the cassette within 80 min. The functionality of the cassette was validated by detecting the presence of a SARS-CoV-2 pseudovirus and three other respiratory pathogens, i.e., *Klebsiella pneumoniae*, *Pseudomonas aeruginosa*, and *Stenotrophomonas maltophilia*. The limit of detection (LoD) for the SARS-CoV-2 pseudovirus was 2.5 copies/μL with both primer sets (*N* gene and *ORF1ab* gene), and the three bacterial species were successfully detected with an LoD of 2.5 colony-forming units (CFU)/μL in 800 μL of swab rinse. Thus, the analyzer developed in this study has the potential to rapidly detect SARS-CoV-2 and other respiratory pathogens on site in a “raw-sample-in and answer-out” manner.

## Introduction

Coronavirus disease 2019 (COVID-19), caused by severe acute respiratory syndrome coronavirus 2 (SARS-CoV-2), was first reported in Hubei Province, China, in December 2019^1–4^. As of June 2nd, 2021, there were 171 million confirmed cases of COVID-19, which has resulted in 3.68 million deaths worldwide^5^. The symptoms of COVID-19 are nonspecific, such as fever, respiratory symptoms, and viral pneumonia^6–8^. Accurate identification of infectious pathogens is vital in achieving precision treatment and limiting viral spread within the population^9–12^. Therefore, there is an urgent need for a clinical diagnostic system that can rapidly detect SARS-CoV-2 and distinguish multiple infectious organisms.

Since the outbreak of COVID-19, the Centers for Disease Control and Prevention (CDCs) of China and the United States have rapidly employed real-time reverse transcription-polymerase chain reaction (RT–PCR) approaches as the “gold standard” for the clinical diagnosis and investigation of suspected cases^13,14^. RT–PCR kits have been developed quickly for the qualitative detection of SARS-CoV-2 in nasopharyngeal swabs, alveolar lavage fluid, sputum, and blood samples^15,16^. Although real-time RT–PCR is sensitive and specific, it typically requires a long turnaround time, specialized laboratory facilities, and skilled technicians. In addition, in standard clinical microbiology laboratories, physically separated locations for sample preparation, reagent formulation, reaction setup, amplification, and detection are required to minimize the cross-contamination risk, which limits its broad application to the current rapid growth and demands of testing the large number of suspected patients, asymptomatic patients, and close contacts^17–19^. Therefore, the U.S. Food and Drug Administration (FDA) has authorized point-of-care (POC) tests for SARS-CoV-2 detection^20^. The World Health Organization offers the following criteria for POC infectious disease test devices: affordable, sensitive, specific, user-friendly, rapid and robust, equipment-free, and delivered to end-users^21–23^. Microfluidics technologies have been demonstrated to enable the integration of multiple laboratory functions into portable, robust, accurate, and sensitive genomic diagnostic devices for deployment at the point-of-care^24–29^.

Since the conceptual proposal of micro total analysis systems by Manz et al.^30^, great efforts have been made to automate and integrate multistep laboratory operations into small chips over the last 30 years^31–33^. The development of microfluidic platforms with raw-sample-to-result capability for infectious disease detection is regarded as an important research direction. In 2011, Liu et al.^34^ developed a disposable microfluidic cassette for the detection of infectious diseases. The cassette integrated the functional steps of viral nucleic acid purification, isothermal amplification, and real-time fluorescence detection into one chamber. However, the requirement of external pumps for reagent introduction and manipulation limits the POC use of this system. Since many respiratory infectious diseases show overlapping symptoms and require distinct therapies, tests that can identify the correct pathogens are of great importance. The GeneXpert platform^35,36^, a pioneering product in the molecular diagnosis of infectious diseases, integrates all the necessary steps, from sample introduction to target detection, but the single reaction chamber in the cartridge limits its ability to detect multiple targets. Practical applications in clinical settings require the processing of complex physiological samples. For instance, when diagnosing respiratory infectious diseases, swab samples are reliable sources of patient microbial content. However, in most examples, the pretreatment of original biological samples is not an integrated function on the chip. FilmArray^37,38^ from BioFire Diagnostics is an advanced molecular diagnostic system that allows for fast and comprehensive multiplex PCR testing. However, an additional device is needed to conduct sample loading or sample preparation, and this system is rather costly, which hinders its widespread use. In all of the aforementioned cases, high-level performances of genetic diagnostics were realized. However, the demand for true sample-in-answer-out systems that require little user intervention, feature a rapid analysis period, and perform multiplexed detection of pathogens has not been fully satisfied.

In the last twenty years, our group has been continually developing microfluidic devices for integrated nucleic acid detection^39–41^. In our previous work, a self-contained fluidic cassette system was constructed to detect multiple bacteria in urine samples^42^. Here, this cassette system was further developed to enable the direct processing of an unprocessed swab sample and completion of a panel of assays to detect SARS-CoV-2 and three bacterial species known or suspected to cause respiratory tract infection, i.e., *Klebsiella pneumoniae* (*K. pneumoniae*), *Pseudomonas aeruginosa* (*P. aeruginosa*) and *Stenotrophomonas maltophilia* (*S. maltophilia*)^43–45^. Once the swab sample is inserted into the cassette, the entire analysis procedure, including swab rinsing, silica bead-based nucleic acid extraction, and 8-plex real-time reverse transcription loop-mediated isothermal amplification (RT-LAMP), is automatically executed in 80 minutes. In this system, the SARS-CoV-2 pseudovirus had a limit of detection (LoD) of 2.5 copies per μL with both primer sets (*N* gene and *ORF1ab* gene), and *K. pneumoniae*, *P. aeruginosa*, and *S. maltophilia* had LoDs of 2.5 colony-forming units (CFU) μL^−1^. The portable analyzer features all necessary assay steps for the simultaneous detection of multiple pathogens from swab samples, is easy to use and is highly suited for the molecular diagnosis of respiratory tract infections on site.

## Materials and methods

### Fabrication of the self-contained cassette

The proposed cassette was intended for the implementation of nucleic acid extraction and RT-LAMP reactions for pathogen analysis; it measured 5 × 5 × 10.5 cm, as schematically depicted in Figure S1. The cassette consists of six chambers for holding samples, reagents and waste. The sample chamber has a polycarbonate (PC) lid fixed with a cut flocked swab (Copan Diagnostics, Brescia, Italy). It can hold up to 2.4 mL of liquid, allowing sufficient buffer to rinse the swab surface for sample resuspension and lysis. It features an interface to the cassette and servers both to load and to treat the swab sample. Each of the other five reagent chambers is covered by a flat PC lid with a venting hole. The cassette contains eight reaction chambers with inlet and venting channels for RT-LAMP or LAMP. Each chamber is sealed by a pressure-sensitive adhesive (PSA) cover slip. At the center of the cassette is a Luer syringe that is connected to one outlet of a channel on the PC rotary valve. The outlet of another channel can be precisely positioned to the inlet on the main body of the cassette. To avoid leakage, a polyurethane washer is sandwiched between the rotary valve and the main body of the cassette. Fluidic control within the cassette is achieved by cooperative manipulation of the self-contained syringe and the rotary valve.

Before use, the corresponding reagents for nucleic acid extraction and RT-LAMP amplification were manually loaded into the chambers of the cassette, and the primer pairs used to detect the different types of pathogens were preloaded and dried in the reaction chambers of the cassette.

### Portable analyzer

The portable analyzer has been described in detail previously^42^. Briefly, the analyzer (21×17×24 cm) performs all the tasks conducted in the cassette, consisting of fluid guidance, magnetic silica bead manipulation, heater control, and fluorescence scanning (Figure S2).

To achieve basic fluid control functions, the syringe piston and the rotary valve are precisely controlled by a stepper motor (28-T6, Shengsida Machinery Equipment, Jiangsu, China) and a digital servo (GDW DS945MG, Shenzhen Huaxiang World Technology, Guangdong, China) in a coordinated pattern with a minimal controllable volume of 1 μL. To manipulate the magnetic beads, a self-locked solenoid (ZHK-0521, ZONHEN Electric Appliances, Shenzhen, China) is triggered to adjust the distance between the permanent magnet and the cassette. For enrichment of the magnetic beads, the magnet is pulled close to the bottom of the cassette when the mixed solution with magnetic beads is slowly passed through the center of the rotary valve. To resuspend the collected magnetic beads, the magnet is pushed away from the cassette, and the syringe is rapidly pushed and pulled. To control the reaction temperature in chambers of the cassette, the heating unit contains a silicone rubber heating film (Dongtai Huayang Electrothermal Electrical Apparatus, Shandong, China) and a K-type thermocouple (TT-K-36-SLE, OMEGA, Stamford, CT) regulated by a fuzzy proportional-integral-derivative control algorithm. Real-time fluorescence detection is achieved by scanning the eight reaction chambers using a Y-shaped optical fiber sensor (Nanjing HongZhao, Nanjing, China), which consists of twelve surrounding fibers and one central fiber. The excitation light from a light-emitting diode (LED) (SP-01-B6, Quadica Developments, Alberta, Canada) is filtered through a 455–495 nm bandpass excitation filter (Beijing Bodian Optical, Beijing, China) before being coupled with one of the branched terminals to the twelve surrounding fibers. The emitted light is collected through a central fiber and filtered with a 520–540 nm bandpass emission filter (Beijing Bodian Optical, Beijing, China). Finally, the fluorescence signal is recorded by a photomultiplier tube (PMT) (H9307-02, Hamamatsu, Shizuoka, Japan).

### Preparation of samples

Swab samples were obtained from eight healthy volunteers. A SARS-CoV-2 pseudovirus (FNV-2019-ncov-abEN) provided by Fubio Biological Technology (Suzhou, China) was constructed by a lentiviral vector system (FV115), encapsulating a 6202 bp single-stranded RNA including the full-length *N* gene, *E* gene, and the partial sequence of the *ORF1ab* gene of SARS-CoV-2 with a total length of 1989 bp. The diameter and the overall structure of the pseudovirus (100-120 nm) are both very similar to those of SARS-CoV-2 particles (60-140 nm). The pseudovirus was quantified with a digital PCR system (Target-One, Beijing, China). The SARS-CoV-2 whole genome reference standard (GW-CRPM002) was purchased from GeneWell Biotech (Shenzhen, China). Respiratory pathogens, including *K. pneumoniae* (Gram negative, ATCC 10031), *P. aeruginosa* (Gram negative, ATCC 9027), and *S. maltophilia* (Gram negative, ATCC 17666), were first cultivated in sterilized brain heart infusion (BHI) medium (AoBoXing Bio-Tech, Beijing, China) at 37 °C in a shaker (200 rpm) for 16–18 h. To determine the concentration of bacteria, a small portion of the culture was diluted to an appropriate concentration with distilled water and then enumerated by serial dilution plating on BHI agar medium. Serial dilution of the above viral and bacterial suspensions with phosphate-buffered saline (PBS) (Beijing Solarbio Science & Technology, Beijing, China) was performed for subsequent experiments. *HeLa* cells were obtained from the American Type Culture Collection (ATCC, Manassas, VA) to mimic human epithelial cells. *HeLa* cells were cultured in Dulbecco’s modified Eagle’s medium (DMEM, Invitrogen, Shanghai, China) supplemented with 10% fetal bovine serum (FBS, Invitrogen, Shanghai, China) in a standard cell culture incubator (37 °C and 5% CO_2_) and counted using Countess (Invitrogen, Shanghai, China).

### Benchtop sample preparation protocol

#### Reagents

DNA and RNA extraction and purification from viruses and bacteria were performed using a commercial bacterial nucleic acid extraction kit (AU2001, BioTeke Corporation, Beijing, China). A fluorescent probe-based PCR kit for the detection of the SARS-CoV-2 was purchased from Daan Gene (Guangzhou, China), and qPCR SuperMix Uracil-DNA Glycosylase (UDG) was purchased from Transgen (Beijing, China). The sequences (forward: 5’-GGGGGATCTTCGGACCTCA, reverse: 5’-TCCTTAGAGTGCCCACCCG) that amplified a 956 bp fragment of *P. aeruginosa*^46^ were synthesized by Invitrogen (Beijing, China). The WarmStart LAMP Kit (DNA & RNA) was purchased from New England Biolabs (Beijing, China). Oligonucleotide primers, including two loop primers (LF and LB), two outer primers (F3 and B3), and two inner primers (FIP and BIP) for SARS-CoV-2, the different bacteria and *HeLa* cells, were synthesized by Invitrogen (Beijing, China), the sequences of which are shown in Table S1. All solutions were prepared with DNase/RNase-free distilled water.

#### DNA extraction

For the manual extraction of RNA and DNA, a spiked sample (20 μL) was first added to a 1.5-mL tube containing 710 μL of lysis buffer, 20 μL of proteinase K and 50 μL of lysozyme for 30 min at room temperature under constant shaking for sample lysis. Then, a mixture containing 20 μL of magnetic bead suspension and 180 μL of binding buffer was added to the tube and vortexed for 10 min. Next, the magnetic beads were collected using a magnetic rack, the supernatant was discarded, and a washing buffer (700 μL) was added to suspend and wash the magnetic beads twice for 10 min. After collecting the magnetic beads, 80 μL of ddH_2_O was added to resuspend the magnetic beads for 10 min to release the bound nucleic acids. Finally, the magnetic beads were collected again, and the supernatant was reserved for subsequent analyses.

#### Nucleic acid amplification

The 25-μL PCR mixture prepared for each reaction was composed of 0.5 μL of each primer (10 μM), 12.5 μL of 2×TransStart Green qPCR SuperMix UDG, 6.5 μL of nuclease-free water, and 5 μL of DNA template. PCR was performed in a Bio–Rad CFX96 real-time system (Bio–Rad Laboratories, Shanghai, China). The thermal cycling protocol included an initial activation of UDG at 94 °C for 10 min, followed by 40 cycles at 94 °C for 5 s, 60 °C for 15 s, and 72 °C for 10 s. A 25-μL RT-LAMP assay consisted of 8 U of Bst 2.0 WarmStart DNA polymerase, 0.5 μL of WarmStart RTx reverse transcriptase, 1.4 mM dNTP mix, 6 mM MgSO_4_, 1x isothermal amplification buffer, 0.2 μM each of F3 and B3, 1.6 μM each of FIP and BIP, 0.4 μM each of LF and LB, 0.5 μL of fluorescent dye and 12.5 μL of nucleic acid template. The reaction was performed at 65 °C for 40 minutes with a Bio–Rad CFX96 real-time system. All RT-LAMP assays on the cassette were performed with the same constituents as those described for the 25-μL assay mixture but were scaled to the appropriate volume.

### Operational procedure of the cassette system

**Fig. 1:**
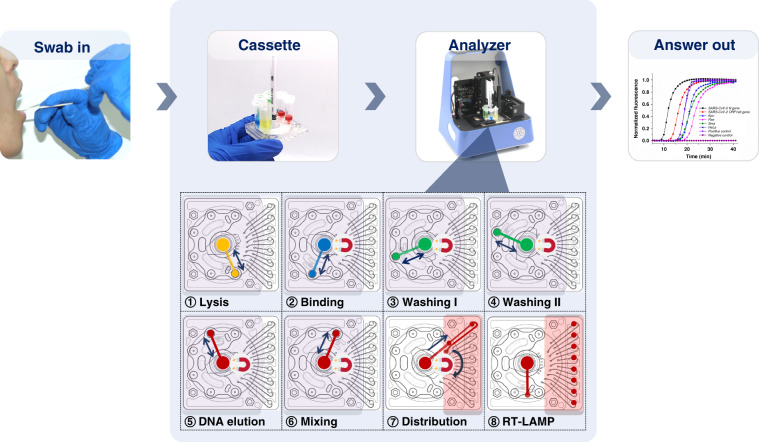
The workflow of the pathogen detection process. After loading a swab specimen into a cassette and inserting the cassette into the instrument, all the following processing procedures are conducted automatically. The entire flow control of the cassette operates in the following sequences: (1) the swab sample is washed using lysis buffer, (2) the DNA binds to magnetic beads, (3–4) DNA-bound magnetic beads are washed twice with washing buffer, (5) the bound DNA is eluted, (6) the DNA eluate is mixed with the RT-LAMP reagent, (7) the mixture is dispensed into eight reaction chambers, and (8) real-time RT-LAMP is performed. After the RT-LAMP reactions, the presence of the pathogens can be determined from the amplification curves of eight reaction chambers

A typical testing workflow and the control program of the cassette system in this study are shown in Fig. 1. After collecting the swab samples, each swab was transferred into the lysis chamber and completely immersed in lysis buffer on the cassette (Figure S3). The cassette was then inserted into the analyzer, and all of the following operations were automatically performed by the analyzer (Video S1). The sequence of the processes is described below.

#### Swab washing and sample lysis

The rotary valve was rotated to switch the inlet of the lysis chamber, after which the swab surface was thoroughly washed with lysis buffer (800 μL) by quickly transferring liquid between the Luer syringe and the lysis chamber for 30 min at a 150 μL/s flow velocity. Sample resuspension and lysis were accomplished after this process.

#### DNA extraction

The on-cassette DNA extraction process was developed from the manual protocol described above. First, 800 μL of the lysed sample was driven into the Luer syringe, after which the rotary valve was rotated clockwise to connect the inlet of the binding chamber and the Luer syringe. Then, the lysed sample was transferred into the binding chamber and mixed with a binding buffer containing magnetic beads (200 μL) for DNA binding for 10 min at a 150 μL/s flow velocity. Subsequently, the mixture (1 mL) was aspirated back into the Luer syringe and then slowly pushed into the binding chamber through the channel inside the rotary valve with a flow rate of 30 μL/s. Simultaneously, the magnetic beads were collected in the bottom of the rotary valve by the magnetic field generated by the magnet located close to the bottom of the rotary valve.

Subsequently, the rotary valve was switched to connect the Luer syringe and the inlet of the first washing chamber, and the magnet was moved away from the rotary valve. Then, the Luer syringe was quickly pushed and pulled several times to resuspend and wash the magnetic beads (700 μL with a rate of 200 μL/s). After the first washing process was complete, the magnet was lifted again to collect the magnetic beads, and the waste buffer in the Luer syringe was discarded into the first washing chamber. The second washing step followed a similar process.

After the washing process was completed, the magnet was moved away from the rotary valve, and the magnetic beads were resuspended in ddH_2_O (80 μL) for 10 min to elute the bound nucleic acid on the magnetic beads. Afterward, the magnetic beads were collected again, and the eluted nucleic acid (80 μL) was aspirated into the Luer syringe. Then, it was mixed with the RT-LAMP master mix (80 μL) in the mixing chamber with the rotary valve connected to the inlet of the mixing chamber.

#### LAMP reactions and detection

Finally, the RT-LAMP mixture was precisely delivered into the eight reaction chambers with the prestored primer pairs. The eight reaction chambers were isolated by a rotary valve that was sealed with a polyurethane washer, eliminating the potential for cross-contamination among the adjacent reaction chambers. The reaction chambers of the cassette were incubated in situ at 65 °C for RT-LAMP, and the fluorescence detection system was driven by a stepper motor to continuously scan and extract the fluorescence signal of each chamber to plot the corresponding amplification curve.

The workflow described above can be easily reprogrammed to meet the needs of other experimental protocols.

## Results and discussion

### Evaluation of on-cassette swab rinse

Our cassette system was designed to detect respiratory pathogens from untreated swab samples. Therefore, maximizing the yield of pathogens adhered to a swab became our first concern. To demonstrate the swab rinsing performance of the device, simulated swab samples were prepared by adding 20 μL of PBS with *P. aeruginosa* (10^4^ CFU μL^−1^) and SARS-CoV-2 pseudovirus (10^5^ copies μL^−1^) before loading into the lysis chamber of the cassette. Then, the swab was transferred into the lysis chamber of the cassette to perform swab washing without the following on-cassette extraction steps. A benchtop manual swab rinse and direct liquid sample lysis without the swab were also conducted for comparison. After purifying the cassette and benchtop nucleic acids with the benchtop protocol described previously, the extracted nucleic acids were quantified using qRT–PCR and qPCR with a Bio–Rad CFX96 instrument. Samples were tested in triplicate.

As shown in Figure S4, no significant differences were found among the three sample pretreatment procedures for SARS-CoV-2 and *P. aeruginosa*. Therefore, on-cassette swab rinsing generated nucleic acid yields similar to those of benchtop manual swab rinsing and direct liquid sample lysis, illustrating that on-cassette swab rinsing is suitable for swab sample pretreatment and lysis.

### Optimization of the on-cassette nucleic acid extraction protocol

The mixing time for each extraction step is essential for nucleic acid extraction for both viral RNA and bacterial DNA and thus was investigated in this study. The SARS-CoV-2 pseudovirus and *P. aeruginosa* were added to 800 μL of lysis buffer at final concentrations of 2.5 × 10^3^ copies μL^−1^ and 2.5 × 10^2^ CFU μL^−1^, respectively. The BioTeke bacterial nucleic acid extraction protocol was as follows: cell lysis time, 30 min; binding time, 10 min; first washing time, 5 min; second washing time, 5 min; and elution time, 10 min. A series of on-cassette nucleic acid extraction experiments with varying extraction time were conducted to determine the optimal extraction protocol. After elution, the extracted nucleic acid was analyzed with a Bio–Rad CFX96 instrument, and the fluorescence curves of RT-LAMP and LAMP reactions were recorded in real time.

**Fig. 2:**
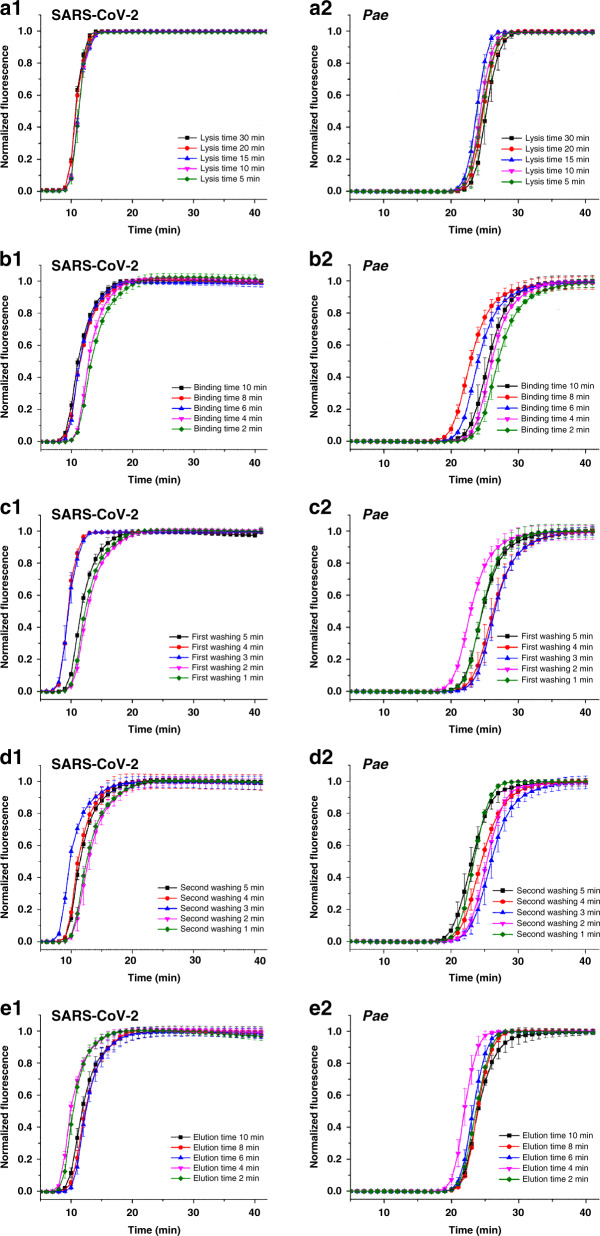
Optimization of the on-cassette nucleic acid extraction protocol. A series of on-cassette extraction experiments for the detection of the *N* gene of SARS-CoV-2 and *P. aeruginosa* with varying extraction time, (a1–2) lysis time, (b1–2) binding time, (c1–2) first washing time, (d1–2) second washing time, and (e1–2) elution time was studied by real-time RT-LAMP or LAMP. The error bars represent the distribution of data from three independent measurements. “Pae” indicates *P. aeruginosa*

The standard amplification curves of the real-time LAMP and RT-LAMP reactions were used to evaluate the extraction efficiencies. For each extraction step, the principle for determining the optimal time is to select a compromise time at which the extraction efficiencies of SARS-CoV-2 and *P. aeruginosa* are comparatively high as well as a relatively short time. When the lysis time was varied (lysis time of 30, 20, 15, 10, and 5 min, with binding, first washing, second washing, and elution time of 10, 5, 5, and 10 min, respectively), as shown in Fig. 2 a1–2, there was no significant change in the detection of SARS-CoV-2, but for the detection of *P. aeruginosa*, the cycle threshold for a lysis time of 15 min was less than that for other time. Therefore, a lysis time of 15 min was used henceforth. When the binding time was varied (binding time of 10, 8, 6, 4, and 2 min, with lysis, first washing, second washing, and elution time of 15, 5, 5, and 10 min, respectively), as shown in Fig. 2 b1–2, binding time of 10, 8 and 6 min was more efficient than 4 and 2 min for detection of SARS-CoV-2 and as efficient for detection of *P. aeruginosa*; 8 min of binding time was the most effective among the five groups. Therefore, the binding time of 8 min was adopted. When the first washing time was modified (first washing time of 5, 4, 3, 2, and 1 min, with lysis, binding, second washing, and elution time of 15, 8, 5, and 10 min, respectively), for detection of SARS-CoV-2, washing time of 2 and 1 min was most efficient; however, for the detection of *P. aeruginosa*, first washing time of 2 and 1 min was least efficient, as shown in Fig. 2 c1–2. To maintain the balance of the first washing efficiency for both SARS-CoV-2 and *P. aeruginosa*, we suggest that the first washing time of 5 min is appropriate for our cassette system. When the second washing time was varied (second washing time of 5, 4, 3, 2 and 1 min, with lysis, binding, first washing, and elution time of 15, 8, 5, and 10 min, respectively), for detection of SARS-CoV-2, second washing time of 3 min was most efficient and washing time of 2 and 1 min was least efficient. However, for the detection of *P. aeruginosa*, second washing time of 5 and 1 min was most efficient, and a second washing time of 3 min was least efficient, as shown in Fig. 2 d1–2. Therefore, to balance the second washing efficiency for both SARS-CoV-2 and *P. aeruginosa*, a second washing time of 5 min was used henceforth. When the elution time was adjusted (elution time of 10, 8, 6, 4, and 2 min, with lysis, binding, first washing, and second washing time of 15, 8, 5, and 5 min, respectively), as shown in Fig. 2 e1–2, an elution time of 4 min was most efficient for both SARS-CoV-2 and *P. aeruginosa*, and thus, an elution time of 4 min was used for our cassette system. Therefore, according to the above experimental results, the optimized on-cassette pathogen nucleic acid extraction protocol was 15 min for lysis, 8 min for binding, 5 min for first washing, 5 min for second washing, and 4 min for elution, and the overall extraction time was approximately 37 min. For further optimization, we can improve the detection sensitivity and shorten the detection time by optimizing the chemical constituents of the nucleic acid extraction kit, increasing the magnetic bead amount, adding an RNA carrier, controlling the incubation temperature of the lysis and elution steps, etc.

### Specificity of the multiplexed detection of pathogens

To verify the viability of multiplexed pathogen detection using our cassette, we performed a few tests with different combinations of the SARS-CoV-2 pseudovirus, bacteria, and *HeLa* cells. Nine groups of samples (800 μL) containing different combinations of the SARS-CoV-2 pseudovirus at a concentration of 2.5 × 10^3^ copies μL^−1^, bacteria at a concentration of 2.5 × 10^2^ CFU μL^−1^ and *HeLa* cells at a concentration of 2.5 × 10^2^ cells μL^−1^ were used to mimic clinical samples. The primer pairs for the *N* gene, *ORF1ab* gene, three types of bacteria, and *RNase* were separately preloaded and dried in the eight reaction chambers before using the cassette, as shown in Figure S5A. In theory, when a sample contains pathogens or *HeLa* cells and they are detected with the cassette, only the reaction chambers containing the corresponding primer pair should yield positive fluorescent signals after the reaction. The tests were repeated at least three times. The results of specificity tests for multiplexed detection of pathogens on the cassette are shown in Table 1 (supplementary Figure S5B1–9). For example, when the sample containing *K. pneumoniae* was inserted into the cassette for detection, only the reaction chamber with a preloaded primer pair specific for *K. pneumoniae* showed a significant increase in fluorescence. Similarly, samples containing other pathogen combinations were also detected as expected. The addition of *HeLa* cells to the samples did not interfere with the detection, which is essential for using our cassette system to detect respiratory pathogens in a human sample. Thus, these results indicate that the cassette can perform accurate and multiplexed detection of pathogens. This test is an illustration, and additional viruses or other pathogens can be detected by replacing the primer pairs and increasing the number of detection chambers if necessary.

**Table 1:** Specificity and multiplicity of on-cassette tests for the detection of viruses and bacteria (Figure S5 shows the amplification results of these tests)

Sample	Primers preloaded in reaction chambers							
	1. *N* gene	2. *ORF1ab*	3. *Kpn*	4. *Pae*	5. *Sma*	6. *RNase*	7. P	8. N
SARS-CoV-2	**+**	**+**	−	−	−	−	**+**	−
*Kpn*	−	−	**+**	−	−	−	**+**	−
*Pae*	−	−	−	**+**	−	−	**+**	−
*Sma*	−	−	−	−	**+**	−	**+**	−
*HeLa*	−	−	−	−	−	**+**	**+**	−
SARS-CoV-2 *& Kpn*	**+**	**+**	**+**	−	−	−	**+**	−
*Kpn & Pae & Sma*	−	−	**+**	**+**	**+**	−	**+**	−
*Kpn & Pae & Sma & HeLa*	−	−	**+**	**+**	**+**	**+**	**+**	−
SARS-CoV-2 *& Kpn & Pae & Sma & HeLa*	**+**	**+**	**+**	**+**	**+**	**+**	**+**	−

“Kpn” indicates *K. pneumoniae*, “Sma” indicates *S. maltophilia*, “+” indicates a positive signal, “−” indicates a negative signal, “P” represents positive control, “N” represents negative control.

### Sensitivity of on-cassette detection

**Fig. 3:**
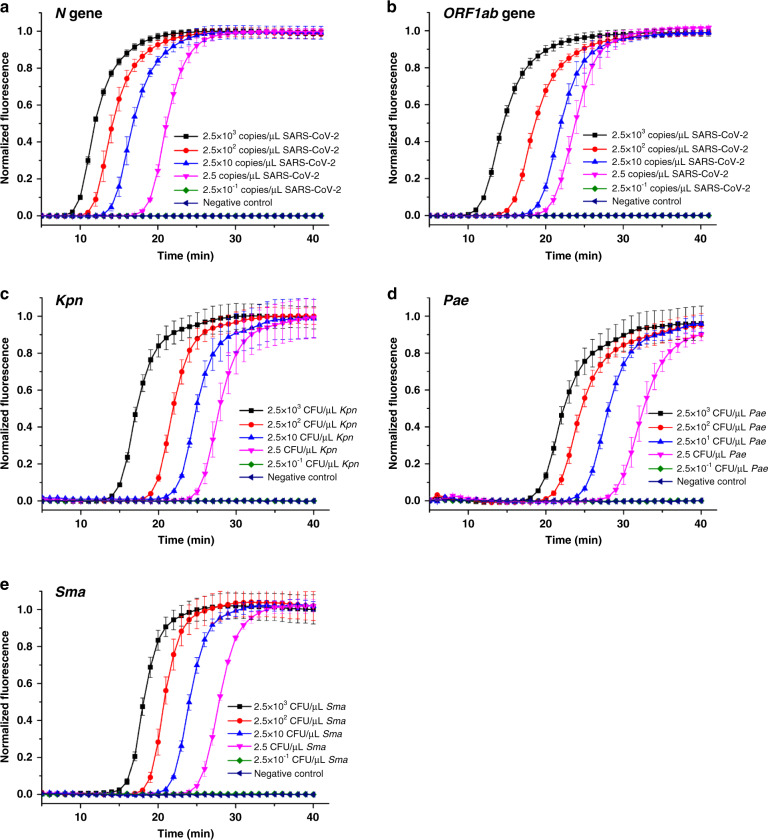
Sensitivity of on-cassette detection of different types of pathogens. **a** *N* gene of SARS-CoV-2, (**b**) *ORF1ab* gene of SARS-CoV-2, (**c**) *K. pneumoniae*, (**d**) *P. aeruginosa*, and (**e**) *S. maltophilia*. The standard deviations of the fluorescence signals from three reactions used for each pathogen are depicted as error bars in all graphs

To evaluate the sensitivity of the cassette-based portable analyzer in the identification of organisms, serial dilutions of the SARS-CoV-2 pseudovirus and bacterial respiratory pathogens were spiked into lysis buffer (800 μL) at varying final concentrations (from 2.5×10^3^ to 2.5 × 10^−1^ copies μL^−1^ and from 2.5 × 10^3^ to 2.5 × 10^−1^ CFU μL^−1^, respectively). The spiked samples were then detected using the analyzer. The amplification curves of SARS-CoV-2 and the three bacterial species at different concentrations are plotted in Fig. 3. For the samples spiked with the SARS-CoV-2 pseudovirus, viral concentrations as low as 2.5 copies μL^−1^ could be amplified to a detectable level using *N* gene primers within 25 min, while it required 30 min using *ORF1ab* gene primers, as shown in Fig. 3a–b. Therefore, the LoD for the SARS-CoV-2 pseudovirus in this cassette was estimated to be 2.5 copies μL^−1^. Similarly, the LoDs for *K. pneumoniae*, *P. aeruginosa*, and *S. maltophilia* were all 2.5 CFU μL^−1^ (Fig. 3c–e). Three replicates were tested for each serial dilution, and the calculated standard deviations are shown with error bars. In addition, a control without template was also run for each test to ensure that the reagents were free of contamination (no false positives were detected). The sensitivity can possibly be enhanced by adding a preamplification step. Efforts are underway to integrate a preamplification chamber into the cassette.

### Evaluation of the cassette system in analysis of swab samples

To investigate the accuracy and clinical applicability of our cassette system, the performance of the direct detection of swab samples is one of the most essential tests. Because clinical samples were unavailable for SARS-CoV-2, we only tested the mimic pathogen. Swab samples from eight healthy volunteers were collected and supplemented with different combinations of pathogens. They were then tested, and the results were obtained with our cassette system. There were three positives and five negatives for SARS-CoV-2 and 2 positives and 6 negatives for *K. pneumoniae*, *P. aeruginosa*, and *S. maltophilia* diagnosed by the cassette system, which was consistent with the previous spiking combinations of the pathogens, as shown in Table S2. The evaluation results demonstrated that our cassette system is suitable for the rapid detection of multiple pathogens from swab samples. To further evaluate the difference between pseudoviruses and SARS-CoV-2, experiments were conducted to compare the RNA of pseudoviruses with the complete genome reference standards of SARS-CoV-2 by RT-LAMP. The cycle thresholds of the *N* gene and *ORF1ab* gene between the above two sets of RNA showed no significant differences at a concentration of 200 copies per reaction (Figure S6). In the near future, additional experiments could be carried out to evaluate the characteristics of pseudoviruses and SARS-CoV-2 once we have access to real samples.

## Conclusions

In this study, a fully integrated and portable analyzer was developed for the rapid detection of multiple respiratory pathogens, including a SARS-CoV-2 pseudovirus, from swab samples. All operations, from swab washing and solid-phase nucleic acid extraction to 8-plex real-time RT-LAMP, can be automatically accomplished in this analyzer within 80 min. The on-cassette swab rinse and nucleic acid extraction processes were optimized and compared with manual operations. Three bacterial pathogens and a SARS-CoV-2 pseudovirus were detected in this cassette-based analyzer with good specificity and sensitivity. The LoD of the SARS-CoV-2 pseudovirus was 2.5 copies μL^−1^ with the *N* gene and *ORF1ab* gene primer sets, and an LoD of 2.5 CFU μL^−1^ was determined for *K. pneumoniae*, *P. aeruginosa*, and *S. maltophilia*. Compared with other studies (Table S3), our cassette system has comprehensive advantages in four aspects: (i) direct detection with untreated swab samples; (ii) magnetic bead-based nucleic acid extraction, which is compatible with the extraction and amplification procedures of both viruses and bacteria; (iii) 8-plex target detection ability, which can distinguish SARS-CoV-2 and other respiratory pathogens; and (iv) a fully integrated hand-held device. Therefore, our analyzer has the ability to rapidly and accurately identify SARS-CoV-2 and other respiratory infectious pathogens on site and has great potential to be further developed as a tool for on-site rapid diagnosis of respiratory pathogens.

## Supplementary information

Supplementary information

Automated processing on the cassette system
