# Supplementary material for: Multiplexed detection of respiratory pathogens with a portable analyzer in a “raw-sample-in and answer-out” manner
Source: Microsyst Nanoeng. 2021 Nov 23;7:94. doi: 10.1038/s41378-021-00321-7 (PMC8608563; doi:10.1038/s41378-021-00321-7)
Supplement: Supplementary file 2 — Automated processing on the cassette system [file 41378_2021_321_MOESM2_ESM.docx]

**Supplementary information**

**Multiplexed detection of respiratory pathogens with a portable analyser in a raw-sample-in and answer-out manner**

Nan Li,^a,†^ Minjie Shen,^a,†^ Jiajia Liu,^a^ Li Zhang,^a^ Huili Wang,^a^ Youchun Xu^a, c*^ and Jing Cheng^a, b, c^

^a^ State Key Laboratory of Membrane Biology, Department of Biomedical Engineering, School of Medicine, Tsinghua University, Beijing 100084, China.

^b^ Center for Precision Medicine, West China Hospital, Sichuan University, Chengdu, 610041, China.

^c^ National Engineering Research Center for Beijing Biochip Technology, Beijing 102206, China.

* Correspondence should be addressed to Youchun Xu (xyc2012@tsinghua.edu.cn).

Tel: (86)-10-62796071.

**Supporting information contents:**

**Table S1.** Sequences of the LAMP primers.

**Table S2.** Detection results for swab samples.

**Table S3.** Summary of POC devices for nucleic acid detection of SARS-CoV-2.

**Figure S1.** Schematic of the cassette for the detection of pathogens.

**Figure S2.** Overview of the fully integrated and portable analyser and schematic diagram of its four functional parts in an exploded view.

**Figure S3.** The original swab was cut and placed into the lysis chamber of the cassette.

**Figure S4.** Three swab sample pre-treatment procedures, including on-cassette swab rinse, manual swab rinse and direct liquid sample lysis, were evaluated by qRT-PCR and qPCR.

**Figure S5.** Specificity and multiplicity of on-cassette tests for the detection of pathogens.

**Figure S6.** Comparison between pseudovirus RNA and complete SARS-CoV-2 genome by RT-LAMP.

**Video S1.** Automated processing on the cassette system.

**Table S1.** Sequences of the LAMP primers.

| **Target** | **Primer** | **Sequences (5'-3')** | **Reference** |
| --- | --- | --- | --- |
| *N* gene of SARS-CoV-2 | F3 | AACACAAGCTTTCGGCAG | 1 |
|  | B3 | GAAATTTGGATCTTTGTCATCC |  |
|  | FIP | CGCATTGGCATGGAAGTCACTTTGATGGCACCTGTGTAG |  |
|  | BIP | TGCGGCCAATGTTTGTAATCAGCCAAGGAAATTTTGGGGAC |  |
|  | LF | TTCCTTGTCTGATTAGTTC |  |
|  | LB | ACCTTCGGGAACGTGGTT |  |
| *ORF1ab* gene of SARS-CoV-2 | F3 | TGCTTCAGTCAGCTGATG | 2, 3 |
|  | B3 | TTAAATTGTCATCTTCGTCCTT |  |
|  | FIP | TCGTATACAGGGCTTTTGACATCTATCTTGGAAGCGACAACAA |  |
|  | BIP | TCAGTACTAGTGCCTGTGCCCACAATCGTTTTTAAACGGGT |  |
|  | LF | CTGCACTTACACCGCAA |  |
|  | LB | GTAGCTGGTTTTGCTAAATTCC |  |
| *Kpn* | F3 | ATACAAAAACACCAGTGTAGG |  |
|  | B3 | GCCGCCAGTTTGTTTCAG |  |
|  | FIP | CGTTGAGATTTGCGAAGTACCAAGAATCAAATATGCTGCAAATGTG |  |
|  | BIP | CAAACGCATAATAAGCAGGTGATTTCGGAGGTGATGTTTTCGGTC |  |
|  | LF | TGCCCGGCCATC |  |
|  | LB | ATCATATCGTTCGGCT |  |
| *Pae* | F3 | CAAGGTGTTCATCCACGA |  |
|  | B3 | CGCTCCAGCGCTTTTCC |  |
|  | FIP | ACTCGTCGCCCATCTCGATGGACTGAACGCCGGTAACCA |  |
|  | BIP | GCACGAGAGCAACGAGATGCGTCTGGGCCATGACCAC |  |
|  | LF | TGTAGATCGGCGACATGTG |  |
|  | LB | CATCAGCCATGCCGGGGTC |  |
| *Sma* | F3 | GTATAAGCTTGGAGGTTCCT |  |
|  | B3 | ATGAAGGCGGCCAGATG |  |
|  | FIP | GCGCCAAGAGCTCAATAGTGGCACCGCTGATCAGGGA |  |
|  | BIP | ACAACTAGTCGTTCACCGGTACGAGGCAATTACAAACGCGATG |  |
|  | LF | GATGCCGGGGGCGGAA |  |
|  | LB | ACCCGATGACAAGGGGCT |  |
| *RNase* | F3 | TTGATGAGCTGGAGCCA | 4 |
|  | B3 | CACCCTCAATGCAGAGTC |  |
|  | FIP | GTGTGACCCTGAAGACTCGGTTTTAGCCACTGACTCGGATC |  |
|  | BIP | CCTCCGTGATATGGCTCTTCGTTTTTTTCTTACATGGCTCTGGTC |  |
|  | LF | ATGTGGATGGCTGAGTTGTT |  |
|  | LB | CATGCTGAGTACTGGACCTC |  |

**Table S2.** Detection results for swab samples.

|  | Pathogen added | | | | Testing results of the cassette system | | | | | | |
| --- | --- | --- | --- | --- | --- | --- | --- | --- | --- | --- | --- |
|  | SARS-CoV-2 | *Kpn* | *Pae* | *Sma* | *N* gene | *ORF1ab* | *Kpn* | *Pae* | *Sma* | *RNase* |  |
| 1 |  |  |  |  | - | - | - | - | - | + |  |
| 2 | √ |  |  |  | + | + | - | - | - | + |  |
| 3 |  | √ |  |  | - | - | + | - | - | + |  |
| 4 | √ |  |  |  | + | + | - | - | - | + |  |
| 5 |  |  | √ |  | - | - | - | + | - | + |  |
| 6 |  |  |  | √ | - | - | - | - | + | + |  |
| 7 | √ | √ |  |  | + | + | + | - | - | + |  |
| 8 |  |  | √ | √ | - | - | - | + | + | + |  |

Note: “√” represents addition of the corresponding pathogen to the swab sample, “+” represents the positive result, “-” represents the negative result.

**Table S3.** Summary of POC devices for nucleic acid detection of SARS-CoV-2.^5-9^

|  | **Platform** | **Sample type** | **Microfluidic platform** | **Sample preparation** | **Analytical method** | **Sensitivity (copies/μL)** | **Multiplex** | **Detection time (min)** | **Size and weight of devices** |
| --- | --- | --- | --- | --- | --- | --- | --- | --- | --- |
|  | Our system | Origin swab | Self-contained syringe and rotary valve | Magnetic bead-based nucleic acid extraction | RT-LAMP | 2.5 | 8-plex | 80 | 21 × 17 × 24 cm^3^, 3 kg |
| 1 | Tian et al^10^ | 4 μL of sample | Centrifugal disk | Chemical lysis | RT-LAMP | 0.5 | 1-plex | 70 | 36 × 61.6 × 52.6 cm^3^ |
| 2 | Soares et al^11^ | 1 μL of sample | Centrifugal disk | Heat inactivation | RT-LAMP | 10 | 1-plex | 60 | 14 × 23 × 8 cm^3^ |
| 3 | Xiong et al^12^ | 12 μL of nucleic acids template | Centrifugal disk | Manual nucleic acid extraction | RT-LAMP | 10 | 8-plex | 40 | 28 × 20 × 13.5 cm^3^ |
| 4 | Xing et al^13^ | 0.4 mL of sample | Centrifugal disk | Silica membrance-based extraction | RT-RPA+LAMP | 0.15 | 5-plex | 45 | 48.5 × 45.2 × 33.1 cm^3^, 25 kg |
| 5 | Davidson et al^14^ | 25 μL of diluted saliva | Paper-based device | Without preprocessing | RT-LAMP | 200 | 1-plex | 60 | -- |
| 6 | Yang et al^15^ | 10 μL of sample | Lateral flow strip | Off-chip one-step releaser | RT-LAMP | 0.5 | 4-plex | 120 | -- |
| 7 | Garneret et al^16^ | 100 μL of sample | Glass fiber-based device | Membrane based extraction | RT-LAMP | 1 | 2-plex | 80 | -- |
| 8 | Huang et al^17^ | 0.8 mL of sample | Movable covers and a series of diaphragm valves | Solid-phase extraction | RT-PCR | 1 | 32-plex | 120 | 39.1 × 25.4 × 16.3 cm^3^, 11 kg |
| 9 | Chen et al^18^ | 0.1 mL of sample | Droplet magnetofluidic cartridge | Magnetic bead-based nucleic acid extraction | CRISPR- Cas12a-Assisted RT-RPA | 1 | 2-plex | 30 | 12.7 × 13.4 × 8.4 cm^3^ |
| 10 | Agrawal et al^19^ | 5 μL of saliva sample | Gravity driven and air-driven valves | Heat inactivation | RT- LAMP+ Cas13 | 40 | 2-plex | 35 | -- |
| 11 | Yin et al^20^ | 30 mL of water sample | Off-chip syringe and manual operation | Flinders Technology Associates membrane | RT-RPA+LAMP | 0.1 | 4-plex | 60 | -- |
| 12 | Ramachandran et al^21^ | 10 μL of sample | Electric field control | Isotachophoresis | RT- LAMP+ Cas12 | 10 | 3-plex | 40 | -- |
| **Commercial devices for detection of SARS-CoV-2 approved by FDA** | | | | | | | | | |
|  | **Platform** | **Sample type** | **Microfluidic platform** | **Sample preparation** | **Analytical method** | **Sensitivity (copies/μL)** | **Multiplex** | **Detection time (min)** | **Size and weight of devices** |
| 13 | Cepheid  GeneXpert^22^ | 1 mL of sample | Self-contained syringe barrel and valve body | Lysis by sonication | RT-PCR | 0.25 | 4-plex | 45 | 10.16 × 30.48 × 29.72 cm^3^, 8.2 kg |
| 14 | BioFire Filmarray^23^ | 200 μL of sample | Pouch format with nest array spots | Magnetic bead-based nucleic acid extraction | Nest PCR | 0.33 | 102-plex | 60 | 39.1 × 25.4 × 16.3 cm^3^, 8.2 kg |
| 15 | Accula  Mesa  Biotech^24^ | 60 μL of sample | Gravity driven, film valve and lateral flow strip | Heat inactivation and chemical lysis | RT-PCR | 0.15 | 2-plex | 30 | -- |
| 16 | ePlex GenMark  Diagnostics^25^ | 200 μL of sample | Electrowetting digital  microfluidics | Magnetic bead-based nucleic acid extraction | RT-PCR | 100 | 20-plex | 120 | 59 × 48 × 54 cm^3^, 49 kg |
| 17 | ARIES  Luminex^26^ | 200 μL of sample | Self-contained pump and valve | Magnetic bead-based nucleic acid extraction | RT-PCR | 180 | 6-plex | 120 | 39.1 × 61.2 × 95 cm^3^, 63.5 kg |
| 18 | Simplexa DiaSorin Molecular^27^ | 50 μL of sample | Centrifugal disk | Without preprocessing | RT-PCR | 0.5 | 4-plex | 60 | 30 × 20 × 30 cm^3^, 7.7 kg |
| 19 | QIAstat-Dx QIAGEN GmbH^28^ | Swab sample | Air pump and self-contained valve | Membrane based  nucleic acid extraction | RT-PCR | 0.5 | 48-plex | 60 | 23.4 × 32.6 ×51.7 cm^3^, 21 kg |
| 20 | cobas Liat Roche^29^ | 200 μL of sample | Compressing the flexible tube segment | Magnetic bead-based nucleic acid extraction | RT-PCR | 5.4 | 6-plex | 20 | 24.1 × 11.4 ×19 cm^3^, 3.76 kg |

**Figure S1.** Schematic of the cassette for the detection of pathogens. (A) An exploded view of the cassette; 1: swab stick, 2: lid, 3: reagents and waste storage chambers, 4: nuts, 5: main body of the cassette, 6: polyurethane washer, 7: screws, 8: Luer syringe, 9: PSA cover slip, 10: rotary valve and 11: cassette base. (B) Photograph of the assembled cassette in its storage state. The chambers are filled with dyes for better visibility. a: lysis chamber, b: binding chamber, c: first washing chamber, d: second washing chamber, e: elution chamber and f: mixing chamber. In (1) and (2), the top and side views of the eight reaction chambers are shown, respectively. (C) Fluidic control principle of the cassette. There is a channel (in bule) on the rotary valve, one outlet of the channel is connected with the Luer syringe, and the other outlet is used to connect each liquid chamber inserted in the main body of the cassette. Therefore, the rotary valve can switch the connection of the Luer syringe in the center and the liquid chambers located outside. Red arrows show the moving direction of the Luer syringe and the green arrow shows the moving direction of the rotary valve.


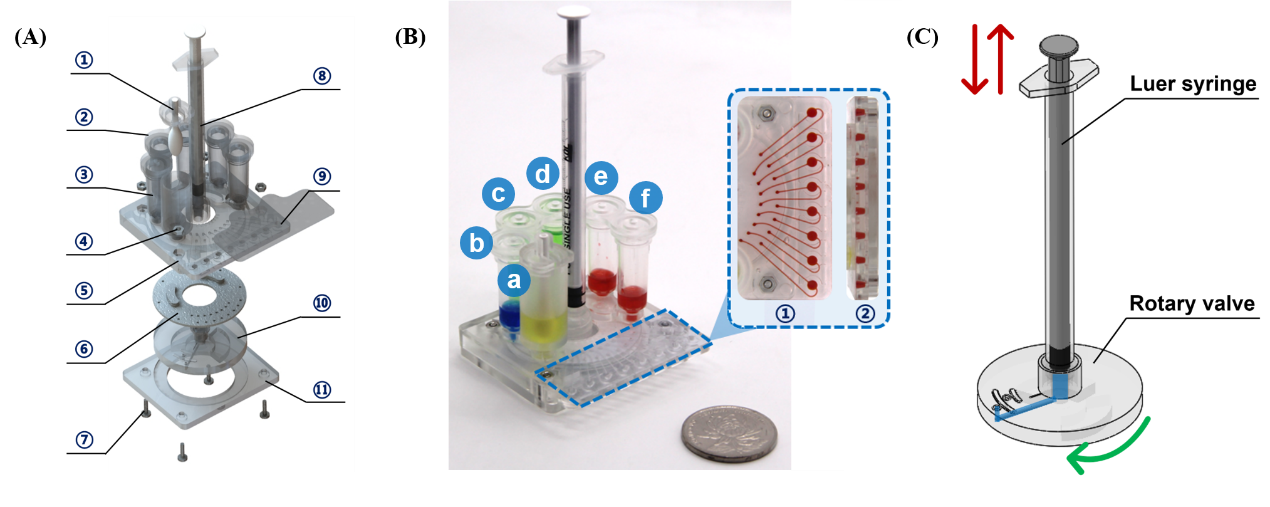


**Figure S2.** Overview of the fully integrated and portable analyser and schematic diagram of its four functional parts in an exploded view.


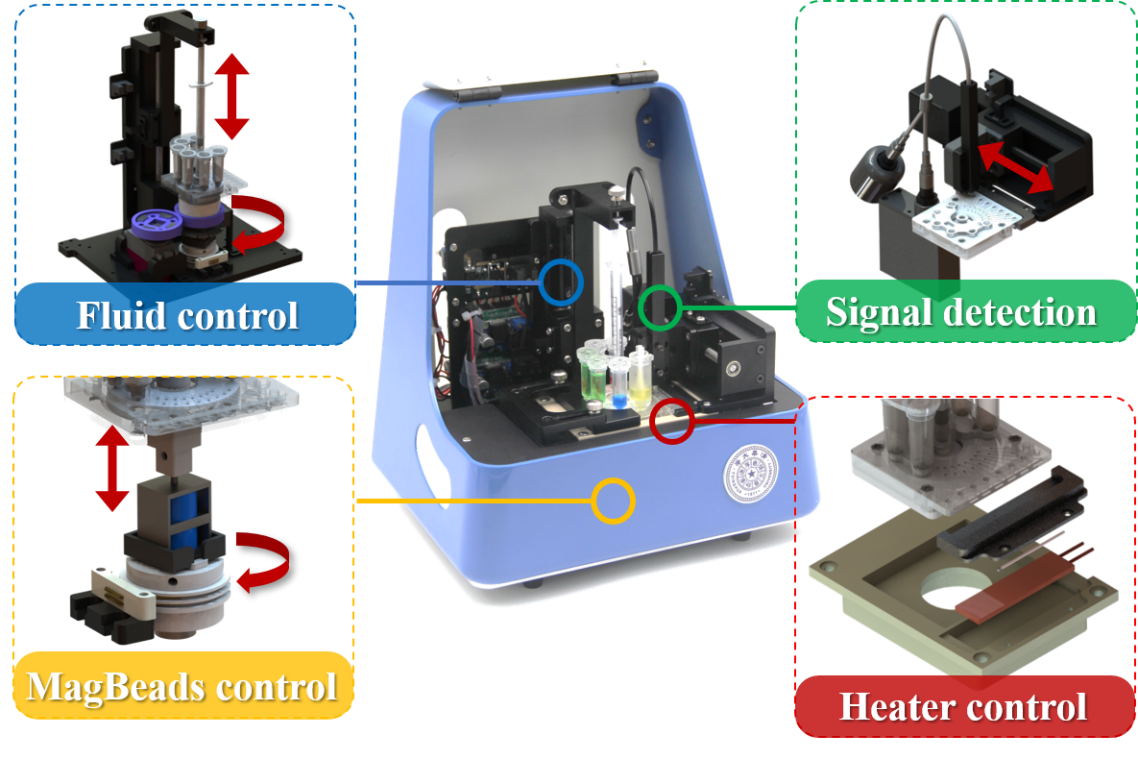


**Figure S3.** The original swab was cut and placed into the lysis chamber of the cassette. 1) Collecting the swab sample, 2) breaking the swab by the lid of the sample chamber, 3) transferring the swab into the lysis chamber, and 4) assembling the cassette. The reagents are represented by differently coloured dyes.


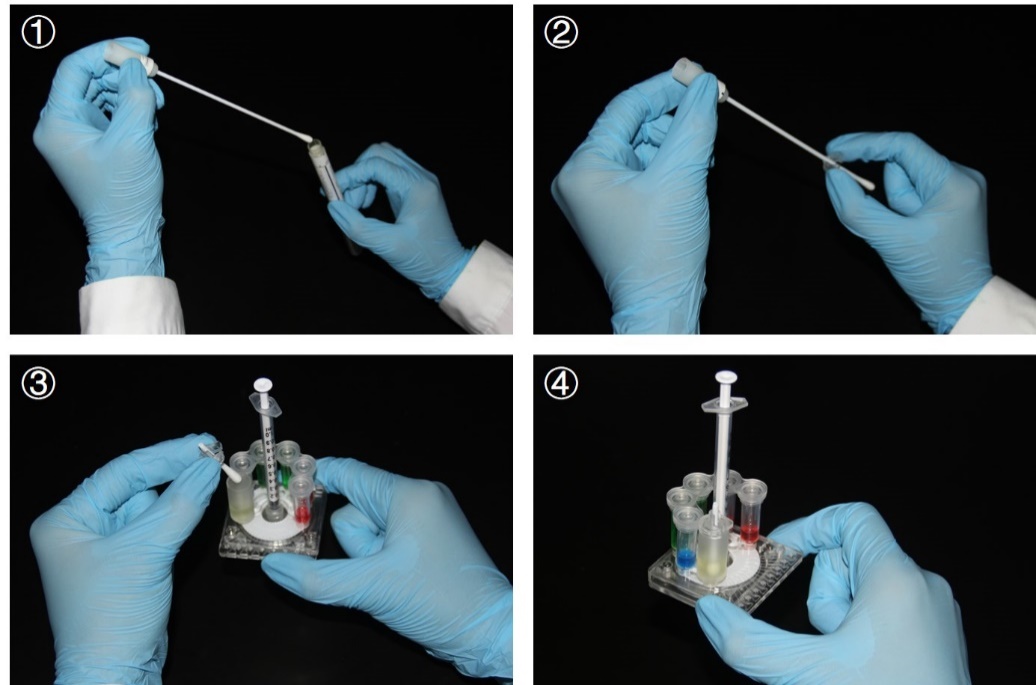


**Figure S4.** Three swab sample pre-treatment procedures, including on-cassette swab rinse, manual swab rinse and direct liquid sample lysis, were evaluated by qRT-PCR and qPCR. (A) SARS-CoV-2 pseudovirus and (B) *P. aeruginosa*.


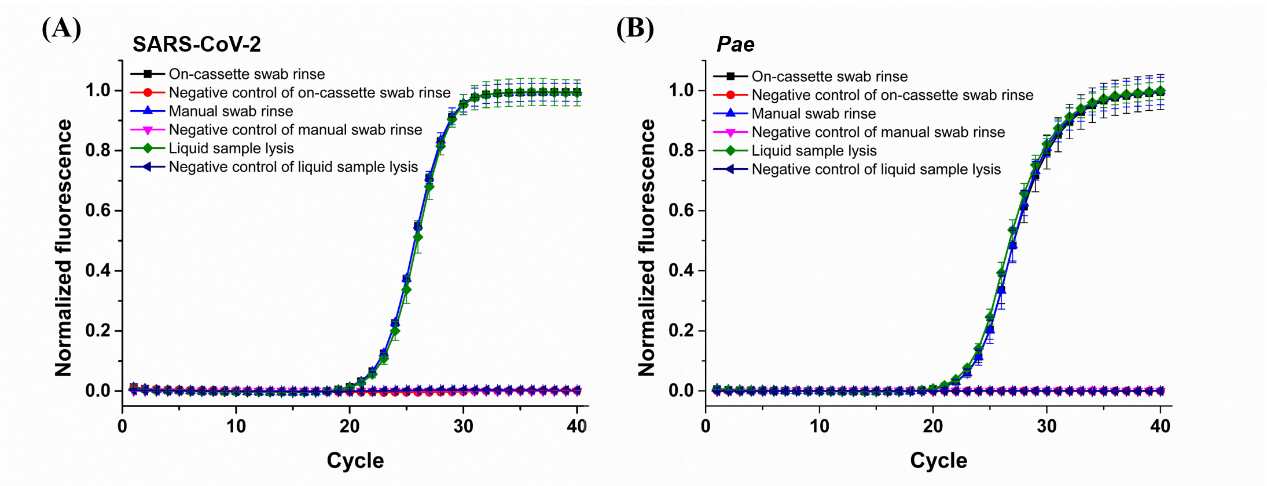


**Figure S5.** Specificity and multiplicity of on-cassette tests for the detection of pathogens. (A) Primers were preloaded in eight reaction chambers. (B1-9) Different combinations of SARS-CoV-2 pseudovirus and three different bacteria were used for specificity tests.


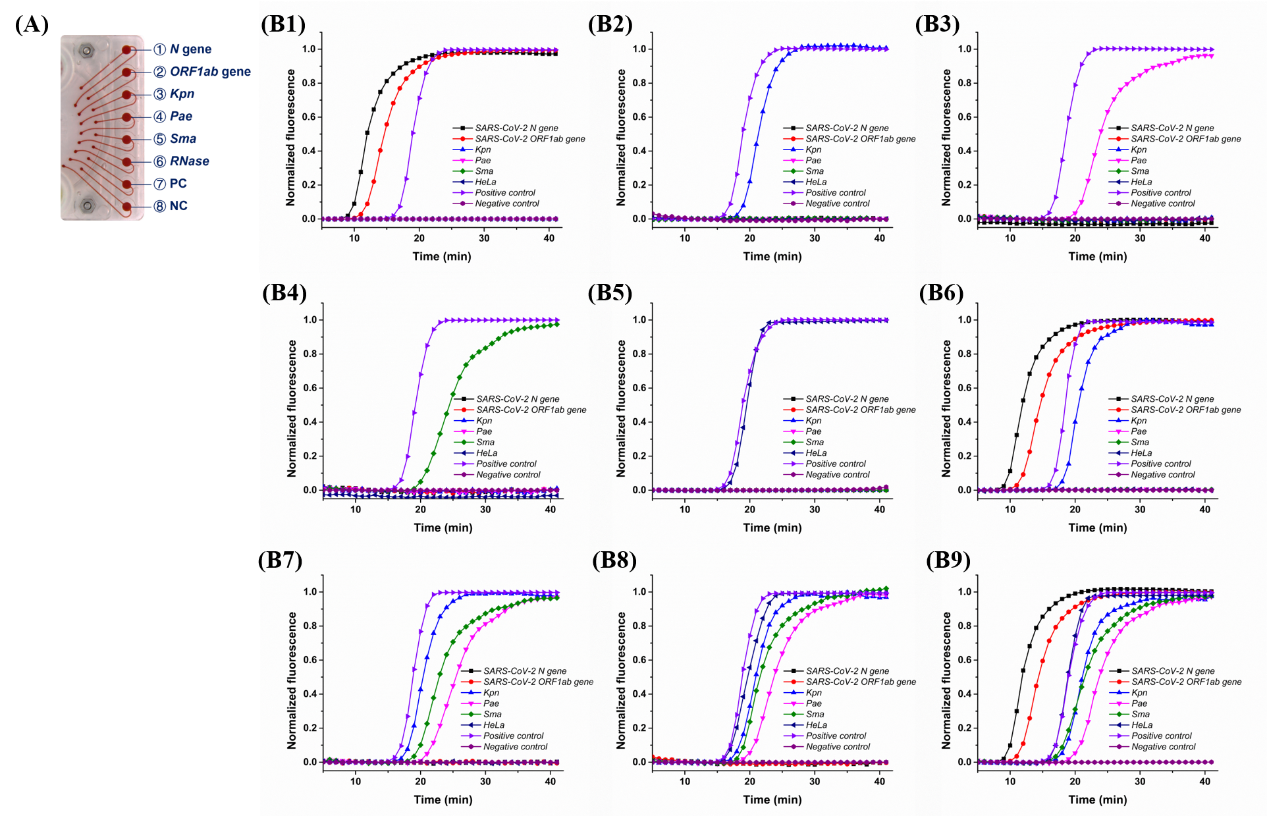


**Figure S6.** Comparison between pseudovirus RNA and complete SARS-CoV-2 genome by RT-LAMP. (A) *N* gene and (B) *ORF1ab* gene. All tests were repeated in triplicate.


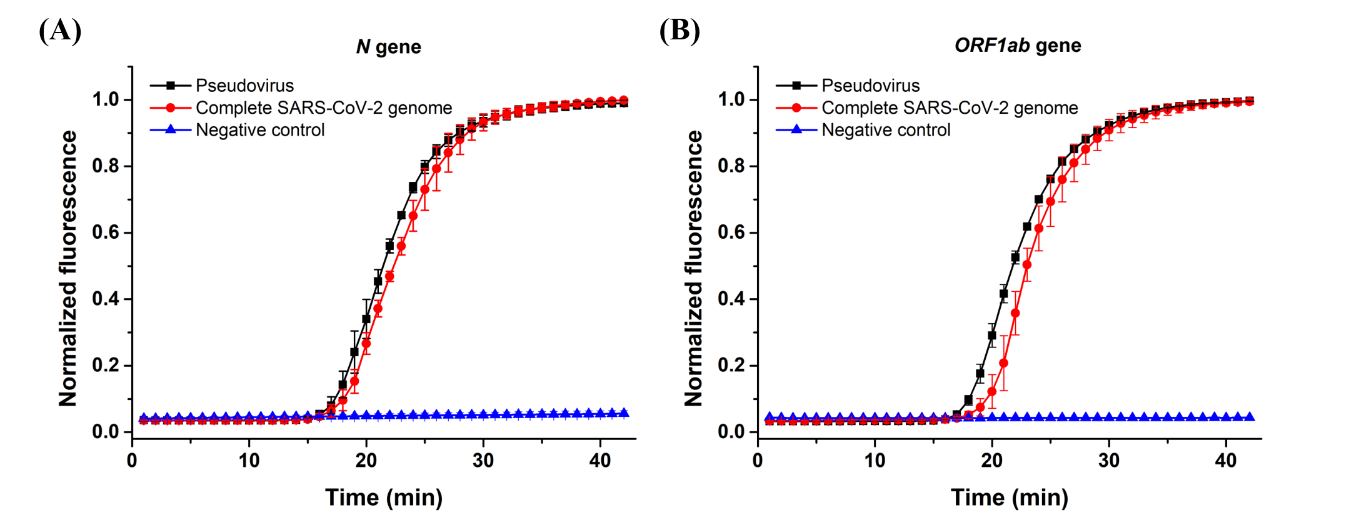


**Video S1.** Automated processing on the cassette system. The video shows the on-cassette fluid control steps, and the sample and reagents are represented by differently coloured dyes.

**References**

1. Broughton JP, Deng X, Yu G, et al, CRISPR-Cas12-based detection of SARS-CoV-2, *Nature Biotechnology*, **38**, 870-874 (2020).

2. El-Tholoth M, Bau HH, Song J, A single and two-stage, closed-tube, molecular test for the 2019 Novel Coronavirus (COVID-19) at home, clinic, and points of entry. Preprint, *ChemRxiv*, (2020). DOI: 10.26434/chemrxiv.11860137.v1.

3. García-Bernalt Diego J, Fernández-Soto P, Domínguez-Gil M, et al, A simple, affordable, rapid, stabilized, colorimetric, versatile RT-LAMP assay to detect SARS-CoV-2, D*iagnostics*, **11**, 438 (2021).

4. Curtis KA, Morrison D, Rudolph DL, et al, A multiplexed RT-LAMP assay for detection of group M HIV-1 in plasma or whole blood, *Journal of Virological Methods*, **255**, 91-97 (2018).

5. Qin Z, Peng R, Baravik IK, Liu X, Fighting COVID-19: integrated micro- and nanosystems for viral infection diagnostics, *Matter*, **3**, 628-651 (2020).

6. Song Q, Sun X, Dai Z, et al, Point-of-care testing detection methods for COVID-19, *Lab on a Chip*, **21**, 1634-1660 (2021).

7. Tarim EA, Karakuzu B, Oksuz C, et al, Microfluidic-based virus detection methods for respiratory diseases, *Emergent Materials*, 1-26 (2021).

8. Basiri A, Heidari A, Nadi MF, et al, Microfluidic devices for detection of RNA viruses, *Reviews in Medical Virology*, **31**, 1-11 (2021).

9. Wang C, Liu M, Wang Z, et al, Point-of-care diagnostics for infectious diseases: From methods to devices, *Nano Today*, **37**, 101092 (2021).

10. Tian F, Liu C, Deng J, et al, A fully automated centrifugal microfluidic system for sample-to-answer viral nucleic acid testing, *Science China. Chemistry*, 1-9 (2021).

11. Soares RRG, Akhtar AS, Pinto IF, et al, Sample-to-answer COVID-19 nucleic acid testing using a low-cost centrifugal microfluidic platform with bead-based signal enhancement and smartphone read-out, *Lab on a Chip*, **21**, 2932-2944 (2021).

12. Xiong H, Ye X, Li Y, et al, Rapid differential diagnosis of seven human respiratory coronaviruses based on centrifugal microfluidic nucleic acid assay, *Analytical Chemistry*, **92**, 14297-14302 (2020).

13. Xing W, Wang J, Zhao C, et al, A highly automated mobile laboratory for on-site molecular diagnostics in the COVID-19 pandemic, *Clinical Chemistry*, **67**, 672-683 (2021).

14. Davidson JL, Wang J, Maruthamuthu MK, et al, A paper-based colorimetric molecular test for SARS-CoV-2 in saliva, *Biosensors & Bioelectronics: X*, **9**, 100076 (2021).

15. Yang M, Tang Y, Qi L, et al, SARS-CoV-2 point-of-care (POC) diagnosis based on commercial pregnancy test strips and a palm-size microfluidic device, *Analytical Chemistry*, (2021). DOI: 10.1021/acs.analchem.1c01829.

16. Garneret P, Coz E, Martin E, et al, Performing point-of-care molecular testing for SARS-CoV-2 with RNA extraction and isothermal amplification, *PLoS One*, **16**, e0243712 (2021).

17. Huang E, Wang Y, Yang N, et al, A fully automated microfluidic PCR-array system for rapid detection of multiple respiratory tract infection pathogens, *Analytical and Bioanalytical Chemistry*, **413**, 1787-1798 (2021).

18. Chen FE, Lee PW, Trick AY, et al, Point-of-care CRISPR-Cas-assisted SARS-CoV-2 detection in an automated and portable droplet magnetofluidic device, *Biosensors & Bioelectronics*, **190**, 113390 (2021).

19. Agrawal S, Fanton A, Chandrasekaran SS, et al, Rapid, point-of-care molecular diagnostics with Cas13, *medRxiv: the preprint server for health sciences*, (2021). DOI: 10.1101/2020.12.14.20247874.

20. Yin K, Ding X, Xu Z, et al, Multiplexed colorimetric detection of SARS-CoV-2 and other pathogens in wastewater on a 3D printed integrated microfluidic chip, *Sensors and Actuators. B, Chemical*, **344**, 130242 (2021).

21. Ramachandran A, Huyke DA, Sharma E, et al, Electric field-driven microfluidics for rapid CRISPR-based diagnostics and its application to detection of SARS-CoV-2, *Proceedings of the National Academy of Sciences of the United States of America*, **117**, 29518-29525 (2020).

22. Raja S, Ching J, Xi L, et al, Technology for automated, rapid, and quantitative PCR or reverse transcription-PCR clinical testing, *Clinical Chemistry*, **51**, 882-890 (2005).

23. Poritz MA, Blaschke AJ, Byington CL, et al, FilmArray, an automated nested multiplex PCR system for multi-pathogen detection: development and application to respiratory tract infection, *PLoS One*, **6**, e26047 (2011).

24. Hogan CA, Garamani N, Lee AS, et al, Comparison of the accula SARS-CoV-2 test with a laboratory-developed assay for detection of SARS-CoV-2 RNA in clinical nasopharyngeal specimens, *Journal of Clinical Microbiology*, **58**, e01072-20 (2020).

25. Schmitz, Jonathan E, Yi-Wei Tang, The GenMark ePlex®: another weapon in the syndromic arsenal for infection diagnosis, *Future Microbiology*, **13**, 1697-1708 (2018).

26. Lawson SK, Chung D, Das S, et al, Analytical and workflow evaluation of the ARIES sample-to-result molecular assay for Clostridium difficile, *Annals of Clinical and Laboratory Science*, **48**, 168-176 (2018).

27. Bordi L, Piralla A, Lalle E, et al, Rapid and sensitive detection of SARS-CoV-2 RNA using the Simplexa™ COVID-19 direct assay, *Journal of Clinical Virology*, **128**, 104416 (2020).

28. Parčina M, Schneider UV, Visseaux B, et al, Multicenter evaluation of the QIAstat Respiratory Panel-A new rapid highly multiplexed PCR based assay for diagnosis of acute respiratory tract infections, *PLoS One*, **15**, e0230183 (2020).

29. Tanriverdi S, Chen L, Chen S, A rapid and automated sample-to-result HIV load test for near-patient application, *The Journal of Infectious Diseases*, **201**, S52-S58 (2010).
